# Astrocyte endfeet may theoretically act as valves to convert pressure oscillations to glymphatic flow

**DOI:** 10.1098/rsif.2023.0050

**Published:** 2023-07-12

**Authors:** Peter A. R. Bork, Antonio Ladrón-de-Guevara, Anneline H. Christensen, Kaare H. Jensen, Maiken Nedergaard, Tomas Bohr

**Affiliations:** ^1^ Department of Physics, Technical University of Denmark, 2800 Kongens Lyngby, Denmark; ^2^ Center for Translational Neuromedicine, University of Rochester Medical Center, Rochester, NY 14642, USA; ^3^ Center for Translational Neuromedicine, Faculty of Health and Medical Sciences, University of Copenhagen, Copenhagen 2200, Denmark

**Keywords:** cerebrospinal fluid, interstitial fluid, pressure oscillation, pressure valve, glymphatic system

## Abstract

The glymphatic system of cerebrospinal fluid transport through the perivascular spaces of the brain has been implicated in metabolic waste clearance, neurodegenerative diseases and in acute neurological disorders such as stroke and cardiac arrest. In other biological low-pressure fluid pathways such as in veins and the peripheral lymphatic system, valves play an important role in ensuring the flow direction. Though fluid pressure is low in the glymphatic system and directed bulk flow has been measured in pial and penetrating perivascular spaces, no valves have yet been identified. Valves, which asymmetrically favour forward flow to backward flow, would imply that the considerable oscillations in blood and ventricle volumes seen in magnetic resonance imaging could cause directed bulk flow. Here, we propose that astrocyte endfeet may act as such valves using a simple elastic mechanism. We combine a recent fluid mechanical model of viscous flow between elastic plates with recent measurements of *in vivo* elasticity of the brain to predict order of magnitude flow-characteristics of the valve. The modelled endfeet are effective at allowing forward while preventing backward flow.

## 1. Background

Valves in the venous vasculature and peripheral lymphatic systems convert pressure oscillations to directed flows. Still, valve structures have not been identified in either the periarterial or the perivenous spaces of the glymphatic system [1]. Pressure oscillations occur at several timescales in brain fluids, ranging from arterial pulsations several times per second [2] over respirations [3] to slow vasomotion every several seconds [4], and would provide a driving force for bulk flow if coupled to a valve.

Astrocytic vascular endfeet plastered around the cerebral vasculature are connected by gap junctions and may form valves [5]. In this hypothesis, the valves open when artery dilation causes an increased pressure at the astrocytic endfeet and valves close when artery constriction causes a corresponding pressure drop. Since astrocyte endfeet (i) naturally protect the extracellular matrix, (ii) are likely to regulate their stiffness with brain states (e.g. via laminin and aquaporin rafts [6,7]), and (iii) provide a probable route for cerebrospinal influx [8], we propose that astrocyte endfeet may also function as valves to turn pressure oscillations into forward fluid flow.

## 2. The modelled endfoot valve under pressure oscillations

**Figure 1.**
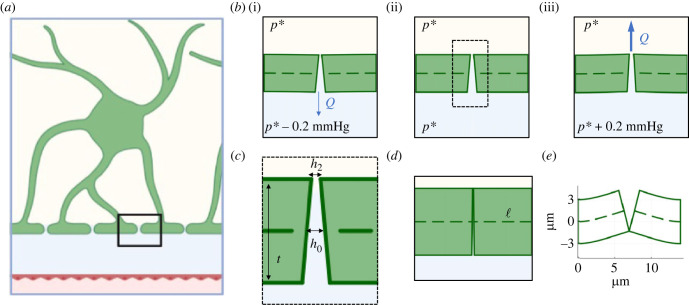
An asymmetry in endfoot gaps may favour inwards over outwards fluid flow. (*a*) An astrocyte (green) near an artery will extend processes with endfeet to cover the periarterial space around the artery (created with BioRender.com). (*b*) Using the *large* parameters (table 1), the endfoot drawn to scale bends only little under the investigated pressure differences of Δ*p* = 0.2 mmHg. (*b*(i)) When the interstitial pressure is greater than perivascular pressure, fluid will be driven out into the perivascular space (with rate *Q*) and endfeet will be pushed together towards closing. (*b*(ii)) When pressures are equal, there will be no flow. (*b*(iii)) When the perivascular pressure exceeds the interstitial pressure, fluid will be driven into tissue. (*c*) The magnified endfoot gap has mid-height of *h*_0_, narrowest height *h*_2_, and thickness *t*. (*d*) Drawn to scale, the *small* endfoot has a relatively small slit opening facing the interstitial side compared with the radius of the endfoot (dashed line, length *ℓ*). (*e*) At larger positive pressures (Δ*p* = 3.4 mmHg), the *large* endfoot gap closes and prevents a fast inflow of cerebrospinal fluid to the interstitial space.

The recent model of viscous flow in a slit between two elastic plates by members of our team, Christensen and Jensen [9], provides a natural minimal framework, figure 1. In their set-up, two flexible plates have a narrow slit between them, like astrocyte endfeet gaps. A key assumption is that the thickness of the plates is much greater than the distance between them. Though precise *in vivo* measurements of astrocyte endfeet dimensions are unfortunately unavailable, they can be estimated from 2-photon imaging of vascular cross-sections (such as those by Enger *et al*. [10]) and *ex vivo* histology (such as that by Wang *et al*. [8]) (table 1). Even considering the wide margins of measurement uncertainty, the endfeet circumference is much greater than their thickness which is much greater than their separation. Though their short radius approaches the limits of its assumptions, this simplified two-dimensional mathematical model is an effective approach to reasoning about asymmetric flow between endfeet (see electronic supplementary material, information).

**Table 1.** Summary of model parameters.

parameter	symbol	*small*	*large*	reference
gap mean height	*h*_0_	0.04 µm	1.0 µm	Wang *et al.* [8]
gap narrow height	*h*_2_	0.02 µm	0.5 µm	
endfoot thickness	*T*	2 µm	6 µm	Enger *et al.* [10]
endfoot radius	*ℓ*	1.5 µm	6.5 µm	Wang *et al.* [8]
endfoot perimeter	*W*	10 µm	40 µm	Wang *et al.* [8]
fluid viscosity	*η*	0.693 × 10^−4^ Pa s	0.693 × 10^−4^ Pa s	Mestre *et al.* [2]
Poisson's ratio	*ν*	0.5	0.5	Goriely *et al.* [11]
Young's modulus	*E*	2.65 ± 0.55 × 10^3^ Pa	2.65 ± 0.55 10^3^ Pa	Green *et al.* [12]

The endfeet may be asymmetric in several ways, but we examine the simplest here. The endfoot gap can be slightly asymmetric, with a narrower slit at the interstitial side than the perivascular side, figure 1. Other asymmetries which may enable valve-function include tethering with interstitial extracellular matrix proteins, internal structure of the cytoskeleton, such as glial fibrillary acidic protein (GFAP) or anchoring to perivascular protein complexes [6]. Here we focus on what is perhaps the simplest realization. Mathematically, we add a single parameter to the original model, the slit height *h*_2_ on the narrower interstitial side, figure 1.

Aside from the static geometry of the endfeet, the model depends on the stiffness of the plate, known as *Young's modulus*, and weakly on the ratio of transverse to axial strain, or *Poisson's ratio*. Despite considerable advances in the measurements of brain mechanical properties, there is a large interval of realistic values possible for both parameters [13–15]. For our calculations here, we will apply both ends of the spectrum of human *in vivo* measurements of macroscopic brain tissue stiffness, table 1, and check results against the softest estimates made (in *in vitro* glia cells [15] see electronic supplementary material, figure S2).

Finally, the model requires a pressure gradient to drive the flow. The pressure oscillations will be most pronounced near arterioles, and we here focus on those periarterial spaces. To best test the valve-function's ability to selectively allow forward flow, we use a pure oscillation with a pressure varying sinusoidally from −0.2 to 0.2 mmHg and with a zero mean based on poroelastic simulations [16] and 2-photon imaging of perivascular spaces [17]. A pressure difference of 0.2 mmHg over the small thickness of the endfoot is large compared with the 1.5 mmHg m^−1^ gradient often considered realistic for cerebrospinal fluid [18], but realistic in light of the local arterial dilation and contraction [16,17,19] and squeeze-flow approximations (see electronic supplementary material, information).

## 3. Astrocyte endfeet may act as valves to convert pressure oscillations to glymphatic flow

**Figure 2.**
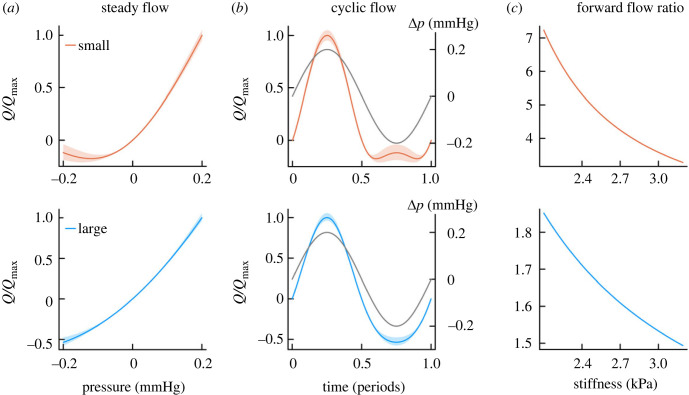
Flexible astrocytes can act as valves to convert pressure oscillations to forward flow. (*a*) In this model, steady flow (*Q)* around *small* (red, top row) and *large* (blue, bottom row) astrocyte endfeet depends asymmetrically on pressure (Δ*p*). Normalized to the maximal flow rate (*Q*_max_) the pressure–flow relationship is similar for *large* and *small* endfeet. (*b*) When exposed to pure pressure oscillations with zero mean, the forward flow is greater than the backward flow for both *small* and *large* endfeet. The *small* endfeet nearly close at maximal backwards pressure, especially when they are soft. Shaded areas show the effect of varying endfoot stiffness within the measurement uncertainty interval (table 1), with softer endfeet allowing greater levels of forward flow than stiff endfeet. (*c*) The effectiveness with which endfeet turn pressure oscillations into forward flow can be quantified as the ratio of forward to backward flow across the pure oscillation, which depends on Young's modulus or stiffness. Both *small* and *large* endfeet can turn pressure oscillations into a driver for forward flow, but *small* endfeet are more effective.

The relationship between flow around the astrocyte endfoot and the pressure gradient is complex and depends on the size and shape of the endfoot, with the size of the gaps between the endfeet being the most important parameter. Due to the considerable measurement uncertainty and the artefacts related to *ex-vivo* histology, we summarize our findings in two scenarios, figure 2*a*. In the *small* scenario, we choose the lower-bounds for gap width, endfoot thickness and radius (figure 2 top row), and in the *large* scenario, we choose the corresponding upper-bounds (figure 2 bottom row). Both *small* and *large* endfeet show the asymmetry, but backflow through the *small* endfeet gaps is more significantly reduced (figure 2*a*).

In figure 2*b*, we show the flow resulting from letting the pressure on the endfeet vary sinusoidally from −0.2 to 0.2 mmHg (assuming quasi-static conditions, see electronic supplementary material, information). As expected from their larger gaps, *large* endfeet provide for greater absolute flow levels. For both *large* and *small* endfeet, the forward flow during positive pressure on the endfoot is greater than the corresponding backward flow during negative pressure.

The ability to turn pressure oscillations into forward flow can be quantified as the ratio of forward to backward flow over the symmetrical pressure oscillation, corresponding to the areas between the horizontal axis and the flow curves in figure 2*b*. Where the *large* endfeet achieve forward flow a couple of times greater than backward, the *small* endfeet achieve forward flow approximately five times greater than backward flow. The greater effectiveness of the *small* endfeet is due to backward flow being more successfully prevented when the narrowest gap height is relatively small. Both *small* and *large* endfeet can in principle achieve perfect effectiveness by closing the gap at the interstitial end completely even at zero pressure (*h_2_* = 0), such that the gap only opens for positive pressures in the perivascular space.

## 4. Discussion

Since several fluid oscillations of considerable amplitude and frequency are present in the live brain, it is important to determine whether any valve mechanisms exist, as the oscillations could then drive directed bulk flow in addition to contributing dispersive clearance [20]. We here drew on recent modelling of fluid mechanics, *ex vivo* quantifications of astrocyte endfeet, and *in vivo* elasticity measurements to argue that endfeet are a realistic valve candidate. We used a geometric asymmetry in the shape of the gap between endfeet, but several sources of asymmetry could promote valve-like behaviour.

Since aquaporins in the endfoot membrane allow faster fluid transport across the membrane, they might contribute to endfoot flexibility [21]. This would connect the circadian localization of AQP4 to the membrane in preparation for sleep [7] with the enhanced fluid flow around the endfoot membrane. However, aquaporins may be anchored to the dystrophin-associated complex via alpha-syntrophin [6] and dystrophin is associated with cell stiffness rather than flexibility [22]. The biomechanical consequences of the aquaporin-associated complexes are important to determine since their regulation may enable a direct mechanical valve function and explain the observed glymphatic dependence on aquaporins.

Efforts to measure the astrocyte endfoot structure along with its mechanical properties *in vivo* are required for progress on this hypothesis. The dynamical deformations necessary for the valve mechanism are relatively small (0.1 µm for *large* endfeet at 0.2 mmHg pressure), or below the *ex vivo* resolution of 2-photon-imaging (approx. 0.2 µm), which in the live brain is further reduced by brain constant movements. Beyond the arteriolar perivascular spaces modelled here, the mechanism may contribute to flow regulation towards the capillary level depending on glia coverage [8,23]. In principle, a reversed asymmetry of the endfoot could also promote efflux rather than influx along arteries as proposed in the iPAD model [24,25]. Until novel imaging approaches can test the valve model or make precise measurements of endfoot mechanical properties, we propose that models of brain fluid flow should consider the possibility that astrocyte endfeet act as valves to convert fluid oscillations to unidirectional glymphatic flow.

## 5. Additional material

The supplementary information with calculations referenced above is available in PDF as Additional File 1. The Calculation Code used to generate the figures is available as a .zip-file collection of Julia code scripts in Additional File 2.

Since *in vivo* measurement of astrocyte endfoot dimensions are lacking and probably vary over orders of magnitude between small and large endfeet [8], we made two parameter scenarios corresponding to the smallest and largest values estimated from the given references. Viscosity is taken as that of water at body temperature and the Poisson's ratio was taken at 0.5, as is common in the literature (with little effect on the calculations [11,26]). Young's modulus was measured *in vivo* in humans with MR elastography in white and grey matter and we here consider the range from mean minus the standard deviation to mean plus the standard deviation [12].

## Data Availability

The data are provided in the electronic supplementary material [27].
